# Skull Vibration-Induced Nystagmus Reveals Hidden Vestibular Asymmetry Dissociated from vHIT, VEMPs and Velocity-Storage-Related Responses: A Clinical–Physiological Case Series and Temporal-Domain Hypothesis

**DOI:** 10.3390/audiolres16050123

**Published:** 2026-08-25

**Authors:** Leonardo Manzari

**Affiliations:** MSA ENT Academy Center, 03043 Cassino, Italy; lmanzari1962@gmail.com; Tel.: +39-3382864625

**Keywords:** skull vibration-induced nystagmus, SVIN, vestibular asymmetry, vHIT, VEMPs, irregular afferents, dimorphic afferents, phase locking, temporal-domain dissociation, velocity storage

## Abstract

**Highlights:**

**What are the main findings?**

- SVIN remained clinically informative across patients with preserved, minimally abnormal, or selectively dissociated canal and otolithic-reflex findings.
- The recurring feature was physiological non-congruence between the vibration-induced ocular response and the canal, otolithic-reflex, or sustained-domain profile.

**What are the implications of the main findings?**

- SVIN should be interpreted as a distinct phase-locked probe of vibration-sensitive vestibular imbalance rather than as a surrogate for vHIT, VEMPs, or sustained-domain testing.
- Dimorphic afferents represent a plausible hypothesis-generating bridge population within the broader irregular, vibration-sensitive vestibular channel.

**Abstract:**

Background/Objectives: Skull vibration-induced nystagmus (SVIN) is a robust sign of vestibular asymmetry, yet its relationship with tests of impulsive canal function, short-latency otolithic reflexes, and sustained visuo-vestibular processing remains incompletely defined. To describe four clinical–physiological cases in which SVIN was dominant or disproportionate relative to the canal, otolithic-reflex, and sustained-domain profiles, and to propose a temporal-domain interpretation of this physiological non-congruence. Methods: Four patients with persistent disequilibrium, episodic vertigo, or selective vestibular-reflex dissociation underwent multidomain neuro-otological assessment, including bedside examination, video head impulse test/head impulse paradigm (vHIT/HIMP), suppression head impulse paradigm (SHIMP) when available, cervical and ocular vestibular-evoked myogenic potentials (cVEMPs and oVEMPs), rotatory or caloric testing when available, and optokinetic or other visuo-vestibular paradigms. SVIN was elicited by 100-Hz mastoid vibration and characterized by direction, dimensionality, stimulation-site dependence, reproducibility, and slow-phase velocity when available. Results: SVIN remained clinically informative across patients with preserved, minimally abnormal, or selectively dissociated canal and otolithic-reflex findings. The recurring feature was not normality of all conventional tests, but physiological non-congruence between the vibration-induced ocular response and the canal, otolithic-reflex, or sustained-domain profile. One patient showed normal six-canal vHIT and symmetric VEMPs despite an exceptionally large fixed-direction SVIN; another showed selective cVEMP absence with preserved oVEMPs and normal vHIT. Atypical multidimensional SVIN coexisted with marked optokinetic directional asymmetry in the adolescent case. Conclusions: SVIN is not a surrogate for vHIT, VEMPs, caloric, rotatory, or velocity-storage-related testing. It should be interpreted as a distinct phase-locked probe of vibration-sensitive vestibular imbalance. Irregular afferents provide the supported physiological framework, whereas dimorphic afferents represent a plausible, hypothesis-generating bridge population capable of contributing to otherwise unexplained clinicophysiological dissociations.

## 1. Introduction

Skull vibration-induced nystagmus (SVIN), commonly evoked by 100 Hz mastoid vibration, is a clinically valuable sign of asymmetric vestibular function. In patients with unilateral vestibular loss, the response is typically abrupt at stimulus onset, maintained during stimulation and rapidly suppressed at stimulus offset, with little or no post-stimulation reversal [1,2].

The increasing availability of multidomain vestibular testing has made clear that vestibular function is not a unitary construct. The video head impulse test (vHIT) explores brief high-acceleration canal-driven vestibulo-ocular reflex gain; cervical and ocular vestibular evoked myogenic potentials (cVEMPs and oVEMPs) explore short-latency otolithic-reflex pathways; caloric and rotatory tests interrogate lower-frequency and sustained vestibular mechanisms; and optokinetic, post-rotatory, visually enhanced vestibulo-ocular reflex (VVOR) and vestibulo-ocular reflex suppression (VORS) paradigms provide information about central visuo-vestibular integration and velocity-storage-related behavior [3,4].

This multidomain approach raises a clinically important question: what does SVIN reveal when the conventional canal, otolithic-reflex, and sustained-domain profile does not predict the vibration-induced ocular response? In daily otoneurological practice, patients with chronic imbalance, persistent dizziness or episodic vestibular symptoms may show clear and reproducible SVIN despite preserved tests in some domains and selective abnormalities in others. Previous studies have already documented non-congruent relationships between vibration-induced nystagmus and caloric, head-shaking, vHIT, and otolithic-reflex measures in partial vestibular disorders, including Ménière’s disease and vestibular neuritis [5,6,7,8,9]. Thus, discordance between SVIN and an individual conventional test is not itself a novel observation.

The present paper describes four illustrative cases selected because SVIN was a dominant or disproportionate finding compared with conventional canal, otolithic-reflex and sustained vestibular tests. The contribution of the present series is to interpret such discordances within an explicitly multidomain and temporal-domain framework, integrating impulsive canal, otolithic-reflex, sustained/velocity-storage-related, visuo-vestibular, and vibration-sensitive responses. In the present context, “hidden” does not mean that all conventional vestibular tests are normal; it refers to a vibration-sensitive asymmetry that is not adequately represented by the overall conventional test profile. Within this framework, SVIN is interpreted as a clinical probe of a vibration-sensitive, irregular-afferent-weighted vestibular ensemble, with dimorphic afferents considered as a plausible candidate bridge population rather than as an established or exclusive substrate.

## 2. Materials and Methods

This study was designed as a retrospective, hypothesis-generating, case-based clinical study derived from routine specialist neuro-otological practice. The clinical observations were obtained from patients evaluated between 16 April and 14 July 2026 at MSA ENT Academy Center, Cassino, Italy, a specialist clinical neuro-otology center dedicated to the assessment, diagnosis, and rehabilitation of vestibular disorders.

All patients were evaluated in a licensed clinical setting as part of standard specialist neuro-otological care. Clinical assessment and patient management were performed by Leonardo Manzari, MD. The data were not obtained from an open database or a third-party source; they were derived from the clinical records of patients evaluated in routine practice and were retrospectively reviewed in anonymized form for development of the present hypothesis-generating clinical framework. No experimental intervention was performed for research purposes. All vestibular tests included in the manuscript had been performed because they were considered clinically appropriate for the diagnostic assessment of the individual patient.

Patients were selected because they showed a reproducible and clinically meaningful vibration-induced nystagmus response that was not fully explained by the pattern of abnormalities on conventional vestibular testing. The key selection feature was therefore not normality of all conventional measures, nor the isolated abnormality of a single test, but a disproportion or physiological non-congruence between the vibration-induced ocular response and the canal, otolithic-reflex, or sustained-domain profile. Direction, stimulation-site dependence, horizontal and vertical components, and coherence with the remainder of the examination were considered in the clinical interpretation. This purposive selection was intended to illustrate selected examples of physiological non-congruence and was not designed to estimate its prevalence, frequency, diagnostic sensitivity, or specificity in the general vestibular population.

All patients underwent detailed clinical history-taking and bedside neuro-otological examination. Bedside assessment included evaluation for spontaneous nystagmus, gaze-evoked nystagmus, positional nystagmus, head-shaking nystagmus, skew deviation when clinically indicated, postural stability, and signs of active benign paroxysmal positional vertigo.

Instrumental assessment was performed according to clinical indication and availability of complete diagnostic data. All vestibular tests were conducted according to standard clinical protocols for each commercially available diagnostic platform and in accordance with the manufacturers’ operating procedures. Because this was a retrospective, hypothesis-generating case series derived from routine clinical practice, test paradigms were selected according to clinical indication rather than applied as a fixed experimental protocol. The principal systems were ICS Impulse with Otosuite Vestibular (Natus Medical Denmark ApS, Taastrup, Denmark) for vHIT, HIMP, SHIMP, VVOR and VORS; ICS Dizcovery videonystagmography (VNG) with Otosuite Vestibular (Natus Medical Denmark ApS, Taastrup, Denmark) for binocular videonystagmography, spontaneous and positional nystagmus, optokinetic testing, gaze, smooth pursuit, skew deviation and SVIN recording; ICS Aircal (Natus Medical Denmark ApS, Taastrup, Denmark) for air caloric testing; an Inventis/Synapsys rotational-chair system with the Nystalyze VNG module (Inventis S.r.l., Padua, Italy) for pendular, sinusoidal, post-rotatory and velocity-storage-related paradigms; and ICS Chartr EP 200 (Natus/Otometrics, Taastrup, Denmark) for cervical and ocular vestibular-evoked myogenic potentials. The exact Otosuite Vestibular and Nystalyze software version numbers were not retained in the retrospective source records and are therefore not reported. Case-specific quantitative parameters are reported in Section 3.

The multidomain test battery was intended to compare distinct temporal expressions of vestibular function. High-frequency/transient canal function was assessed mainly using video head impulse testing, including horizontal and vertical canal evaluation and, when available, the head impulse paradigm (HIMP) and suppression head impulse paradigm (SHIMP). Otolithic-reflex function was assessed using cervical and ocular vestibular-evoked myogenic potentials. Sustained or lower-frequency vestibular processing was assessed using caloric testing, rotational chair paradigms, optokinetic nystagmus (OKN) testing, and other visual-vestibular or velocity-storage-related tests. Dynamic vestibular asymmetry was assessed using head-shaking testing and skull vibration-induced nystagmus when clinically appropriate. For bilateral vHIT/HIMP-SHIMP gain comparisons, normalized asymmetry was calculated as |R − L|/(R + L) × 100. VEMP asymmetry ratios are reported as generated from the clinically recorded amplitude measurements by the electrophysiological system and are not assumed to be interchangeable with the vHIT gain-asymmetry calculation.

For skull vibration-induced nystagmus testing, vibration was delivered using a dedicated multifrequency Synapsys Vestibular Vibrator (Inventis S.r.l., Padua, Italy; product code 10741). The device provides selectable stimulation frequencies of 30, 60, and 100 Hz; all responses included in the present series were obtained at 100 Hz. The system consists of a control unit and a handheld applicator activated by continuous pressure on the handpiece button. The vibrating head was covered with the replaceable biocompatible foam pad supplied by the manufacturer. The applicator was positioned sequentially over the right and left mastoid processes, with stable contact maintained throughout each stimulation epoch. Because this was a retrospective clinical series, neither stimulation duration nor the exact number of repeated trials was imposed prospectively as a fixed experimental protocol. Vibration was maintained long enough to characterize the response onset, sustained morphology, direction, and behavior at stimulus withdrawal, and was repeated when necessary to confirm reproducibility. Source-level review of the original VNG time bases confirmed that individual stimulation epochs were not uniformly restricted to 10 s: where explicit onset/offset markers were available, durations ranged approximately from 10 to 20 s, with epochs extending well beyond 10 s in some recordings. The original VNG time bases were retained for case-level verification. Eye movements were recorded under vision-denied conditions during baseline, stimulation, and immediate stimulus withdrawal.

Eye movements during skull vibration were recorded binocularly under vision-denied conditions using the ICS Dizcovery VNG system (Natus Medical Denmark ApS, Taastrup, Denmark) with Otosuite Vestibular software. The platform supports binocular or monocular recording at 60 or 200 frames/s, high-definition binocular resolution of 1280 × 1024 pixels, and horizontal and vertical eye-tracking ranges of approximately ±30° and ±25°, respectively. Horizontal and vertical slow-phase velocity components were quantified by the software. Baseline spontaneous eye velocity was subtracted from the vibration-evoked response, and traces were reviewed for blink, slippage, tracking loss and other artifacts before clinical interpretation.

Operational positivity required a reproducible nystagmic response that emerged during mastoid vibration, was sustained for the stimulation period, and disappeared or markedly decreased after stimulus withdrawal. A slow-phase velocity (SPV) threshold of >2.5 degrees/s was adopted as an operational criterion together with reproducibility, consistency, correction for spontaneous nystagmus, and exclusion of recording artifacts. Earlier consensus work proposed pathological responses above approximately 2 degrees/s; normative data subsequently showed that responses in healthy subjects are generally absent, low-velocity and inconsistent, and Curthoys summarized that comparable mastoid stimulation in healthy subjects does not produce reliable SVIN above approximately 2.5 degrees/s [5,10,11]. Responses below this threshold could still be described quantitatively but were not used alone to establish test positivity. The direction of the fast phase, stimulation-site dependence, horizontal and vertical components, and temporal profile were recorded. Because the purpose of the study was hypothesis-generating, the cases were analyzed descriptively rather than statistically.

## 3. Results

For uniform cross-case comparison, each case is presented in the same seven-part sequence: clinical phenotype; bedside examination; vHIT/HIMP-SHIMP; cVEMP-oVEMP; sustained/rotatory/optokinetic domain; SVIN morphology and quantitative response; and case-level physiological interpretation. Objective findings are reported first; interpretation is explicitly separated in the final case-level subsection. When a quantitative value was unavailable in the source record, this is stated explicitly rather than inferred. A uniform cross-case summary is provided in Table 1.

### 3.1. Case 1. Chronic Disequilibrium with Disproportionate SVIN and Selective Canal/Optokinetic Abnormalities

**Clinical phenotype.** A 72-year-old woman was evaluated for an approximately 18-month history of persistent postural instability and recurrent lateropulsion, at times severe enough to impair stable walking.

**Bedside examination.** No clinically significant spontaneous nystagmus was observed in the sitting position. Head-shaking elicited only a few beats. Positional testing showed an atypical pattern, with stationary geotropic nystagmus plus a downbeating component in the left lateral position and downbeating nystagmus in the right lateral position.

**vHIT/HIMP-SHIMP.** Horizontal vHIT gain was 0.79 on the left and 1.00 on the right (normalized asymmetry 12%). Vertical gains were 0.54 for the left anterior, 0.69 for the right posterior, 0.91 for the left posterior and 1.01 for the right anterior canal, indicating selective LARP-plane impairment rather than complete unilateral loss. Horizontal SHIMP was preserved (0.91 left, 1.01 right; asymmetry 5%).

**cVEMP-oVEMP.** Cervical and ocular VEMPs had been normal approximately 1 year before the current symptoms. No contemporaneous reassessment was available; these findings were therefore retained as historical information rather than proof of preserved otolithic-reflex function at the present examination.

**Sustained/rotatory/optokinetic domain.** VVOR and VORS were qualitatively preserved. Sinusoidal rotation at 0.25 Hz showed gain 0.80, phase 1.0 degrees and minimal leftward preponderance (0.7 degrees/s). Multi-turn post-rotatory time constants were symmetric (9.2 and 9.9 s). Horizontal OKN was bilaterally reduced (gain 0.28 leftward and 0.20 rightward; mean SPV approximately −7.0 and +5.9 degrees/s).

**SVIN morphology and quantitative response.** Vibration of either mastoid evoked a robust, reproducible and predominantly unidirectional horizontal nystagmus. Right mastoid stimulation produced an averaged-eye peak SPV of 10 degrees/s and mean SPV of 4.9 degrees/s; left mastoid stimulation produced an averaged-eye peak of 8 degrees/s and mean SPV of 3.5 degrees/s, with a largest individual-eye peak of 14 degrees/s. A small vertical component was present but remained much weaker than the horizontal response.

Representative anonymized VNG traces are provided in Supplementary Figure S1.

Case-level physiological interpretation. The profile did not support complete unilateral vestibular loss. Preserved horizontal HIMP/SHIMP and sustained rotational behavior coexisted with selective LARP impairment, reduced OKN and a disproportionately strong vibration-induced response. SVIN therefore revealed a vibration-sensitive asymmetry not adequately represented by horizontal impulsive gain, historical VEMPs or storage-related measures.

### 3.2. Case 2. Persistent Postural Unsteadiness with Normal vHIT, Symmetric VEMPs and Explosive Fixed-Direction SVIN

**Clinical phenotype.** An 82-year-old woman was re-evaluated for persistent postural unsteadiness. Chronic instability, rather than an acute prolonged spinning syndrome, was the dominant complaint.

**Bedside examination.** Baseline ocular activity did not show an organized spontaneous nystagmus comparable in magnitude or morphology to the vibration-evoked response. No active positional syndrome was documented in the available examination record; a quantitative HST result was not available.

**vHIT/HIMP-SHIMP.** Six-canal vHIT was normal, without a clinically meaningful canal-deficit pattern. SHIMP was not required to establish the preserved high-frequency canal profile and was not available in the present dataset.

**cVEMP-oVEMP.** Cervical and ocular VEMPs were bilaterally present and symmetric. Thus, the short-latency vestibulomyogenic outputs showed no conventional lateralizing abnormality.

**Sustained/rotatory/optokinetic domain.** Sinusoidal rotation showed a reduced angular VOR gain of 0.27 at 0.22 Hz, with a phase of 28.0 degrees. Horizontal OKN gain was markedly reduced to 0.03 in both directions. Multi-turn post-rotatory time constants were abbreviated and asymmetric (3.5 and 1.0 s), indicating a separate higher-order impairment of slow visuo-vestibular and velocity-storage-related processing.

**SVIN morphology and quantitative response.** Vibration of both mastoids elicited an exceptionally large, reproducible, fixed-direction left-beating nystagmus. Left mastoid stimulation produced a mixed horizontal–vertical response, with dominant left-beating horizontal and accompanying upbeating vertical components and no appreciable torsion. Right mastoid stimulation produced a predominantly pure horizontal left-beating response, without organized vertical or torsional components. The source record documents a very high-amplitude response; definitive software-derived SPV values were not available in the exported report, and graph-derived estimates were not treated as formal measurements.

**Figure 1:**
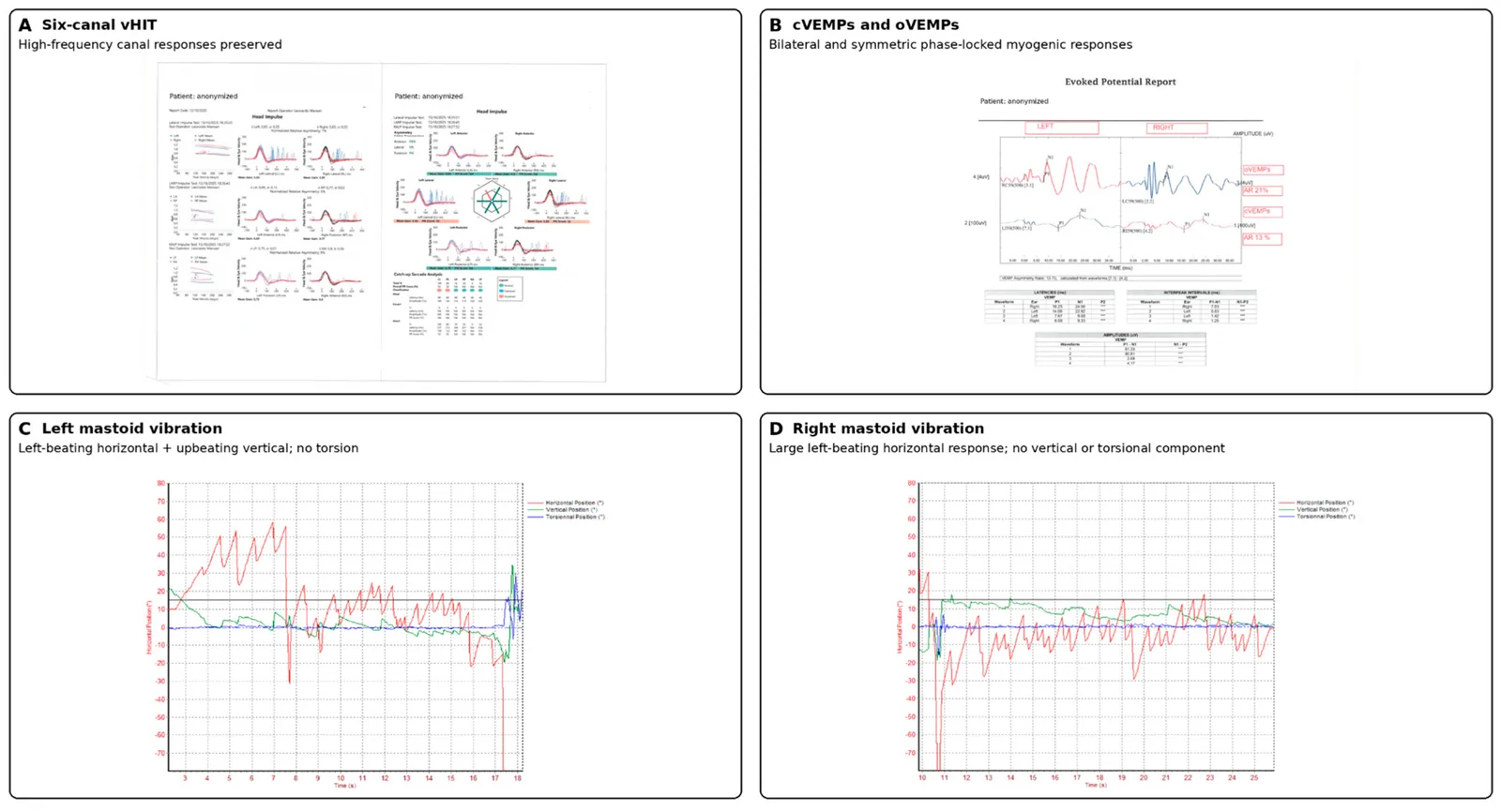
Multidomain vestibular dissociation in Case 2. (**A**) Six-canal video head impulse testing showed preserved high-frequency semicircular canal responses. (**B**) Cervical and ocular vestibular-evoked myogenic potentials were bilaterally present and symmetric. (**C**) Left mastoid vibration elicited a large fixed-direction left-beating horizontal nystagmus accompanied by an upbeating vertical component, without an appreciable torsional component. (**D**) Right mastoid vibration elicited a large left-beating horizontal nystagmus without an organized vertical or torsional component. The figure illustrates a marked intra-phase-locked dissociation: symmetric vestibulomyogenic outputs coexist with an exceptionally asymmetric vibration-induced vestibulo-ocular response. Velocity-storage-related processing represents a separate higher-order temporal-integration level and is not required for the primary expression of stimulus-bound SVIN.

Case-level physiological interpretation. This case demonstrates an intra-phase-locked dissociation: symmetric VEMPs coexist with an explosive asymmetric vestibulo-ocular response to vibration. The abnormal sustained/rotatory profile represents a second, physiologically separate, higher-order temporal-integration abnormality and should neither explain nor reclassify the stimulus-bound SVIN. The multidomain dissociation is illustrated in Figure 1.

### 3.3. Case 3. Adolescent Episodic Vertigo/Headache Phenotype with Atypical Multidimensional SVIN

*Clinical phenotype. A 14-year-old girl was evaluated for recurrent episodes of rotational vertigo described in the retrospective clinical record as lasting up to several days, without a consistent positional or situational trigger, associated with recurrent headache occurring at least twice monthly. Pure-tone thresholds were normal and symmetric (PTA approximately 10 dB HL bilaterally). The available retrospective documentation did not contain sufficient detail to verify all Bárány Society/ICHD-3 vestibular migraine criteria, including the exact duration and migraine-feature association of every individual episode [12].*

**Bedside examination.** No spontaneous or positional nystagmus was observed, and head-shaking did not elicit a pathological response.

**vHIT/HIMP-SHIMP.** High-frequency canal function was preserved. Horizontal vHIT gains were 1.04 on the left and 1.08 on the right (normalized asymmetry 2%), without a definite vertical-canal-deficit pattern. Horizontal SHIMP gains were 0.99 left and 0.98 right.

**cVEMP-oVEMP.** cVEMPs and oVEMPs were bilaterally present, with asymmetry ratios of 17% and 25%, respectively.

**Sustained/rotatory/optokinetic domain.** Sinusoidal rotation at 0.20 Hz showed gain 0.43, phase 2 degrees and minimal leftward directional preponderance. Multi-turn post-rotatory time constants were short but relatively symmetric (5.2 and 6.4 s). The dominant sustained-domain abnormality was severe OKN directional asymmetry: gain 0.35 leftward versus 0.03 rightward at 40 degrees/s.

SVIN morphology and quantitative response. Mastoid vibration evoked an atypical, stimulation-site-dependent multidimensional response. During right mastoid stimulation, both horizontal and vertical components were present; the averaged-eye horizontal response reached a peak SPV of 5 degrees/s (mean 1.8 degrees/s), while the averaged-eye vertical response reached −6 degrees/s (mean −1.3 degrees/s), giving a mixed response with a slightly larger vertical peak. During left mastoid stimulation, the horizontal component was minimal (averaged-eye mean SPV −0.2 degrees/s), whereas the vertical component was clearly predominant (averaged-eye mean SPV −2.7 degrees/s; right-eye vertical peak SPV −10 degrees/s). Explicit onset/offset markers in the original VNG traces documented stimulation epochs of approximately 18–20 s. Thus, the response vector changed with the stimulated mastoid, confirming reproducible multidimensional and stimulation-site-dependent SVIN.

The stimulation-site-dependent horizontal and vertical components, including the original stimulus onset/offset markers, are documented in Supplementary Figure S2.

Case-level physiological interpretation. The combination of preserved canal and otolithic-reflex outputs, marked rightward OKN impairment, recurrent headache, and a stimulation-site-dependent change in the horizontal/vertical SVIN vector did not resemble the classic fixed horizontal SVIN of chronic unilateral vestibular loss. The overall phenotype was considered suggestive of central or network-level visuo-vestibular dysfunction and clinically compatible with a childhood/adolescent vestibular migraine spectrum; however, the retrospective dataset was insufficient to establish formal fulfillment of all diagnostic criteria.

### 3.4. Case 4. Persistent Postural Instability with Selective Otolithic-Reflex Dissociation and Disproportionate SVIN

**Clinical phenotype.** The fourth patient was evaluated for persistent postural instability. The clinical phenotype was dominated by chronic disequilibrium rather than an acute prolonged vestibular syndrome.

**Bedside examination.** Baseline recording showed no clinically meaningful spontaneous nystagmus (horizontal mean SPV approximately −0.4 degrees/s in the right eye and −0.7 degrees/s in the left eye). Head-shaking testing did not elicit a pathological response (mean horizontal SPV approximately −0.7 to −0.8 degrees/s). Thus, the vibration-evoked response was not attributable to a substantial pre-existing spontaneous or post-head-shaking nystagmus.

**vHIT/HIMP-SHIMP.** Six-canal vHIT was normal, without a conventional high-frequency canal-deficit pattern. SHIMP values were not available in the current manuscript dataset.

**cVEMP-oVEMP.** The otolithic-reflex profile was selectively dissociated: the right cVEMP was absent, yielding an asymmetry ratio of 100%, whereas oVEMPs were bilaterally present and symmetric. Thus, the abnormality was not a global otolithic loss but a selective cervical vestibulomyogenic deficit.

**Sustained/rotatory/optokinetic domain.** Horizontal optokinetic responses were reduced. At 40 degrees/s, contemporaneous averaged-eye gains were 0.18 leftward and 0.13 rightward. Earlier recordings also showed a direction-dependent reduction across lower target velocities (20 degrees/s: 0.45 leftward vs. 0.30 rightward; 30 degrees/s: 0.20 leftward vs. 0.07 rightward). Quantitative rotatory and post-rotatory measurements were not available in the present source and were therefore not inferred.

**SVIN morphology and quantitative response.** Skull vibration elicited a reproducible horizontal nystagmus with clear stimulation-site dependence. Left mastoid stimulation produced the larger response, with a right-eye peak SPV of −7 degrees/s and a mean SPV of −3.2 degrees/s and a left-eye peak SPV of −5 degrees/s and a mean SPV of −2.7 degrees/s. Right mastoid stimulation produced a weaker response, with a right-eye peak SPV of −4 degrees/s and a mean SPV of −0.9 degrees/s and a left-eye peak SPV of −2 degrees/s and a mean SPV of −0.8 degrees/s. Explicit onset/offset markers in the original VNG traces documented stimulation epochs of approximately 10–11 s. Baseline spontaneous and post-head-shaking eye velocities remained below the pathological SVIN range. The response therefore showed a reproducible site-dependent asymmetry not explained by spontaneous nystagmus or HST.

Representative source VNG traces documenting the site-dependent horizontal response and stimulus timing are shown in Supplementary Figure S3.

**Case-level physiological interpretation.** This case demonstrates selective dissociation across phase-locked vestibular outputs: an absent right cVEMP with 100% asymmetry coexisted with symmetric oVEMPs, normal six-canal vHIT, and a reproducible quantitatively documented site-dependent SVIN. The vibration-induced ocular response therefore did not simply mirror either canal gain or the isolated cervical vestibulomyogenic deficit. The additional reduction in optokinetic gain represents a separate visuo-vestibular abnormality and further supports a multidomain rather than single-test interpretation.

## 4. Discussion

The central observation of this case series is that skull vibration can evoke a reproducible and clinically meaningful ocular response across patients with preserved, minimally abnormal, or selectively dissociated canal and otolithic-reflex findings. In most cases, SVIN behaved as a stable marker of vibration-sensitive asymmetry; in the adolescent case, its multidimensional morphology and marked optokinetic directional asymmetry suggested additional network-level shaping. The recurring feature was physiological non-congruence between the vibration-induced ocular response and the canal, otolithic-reflex, or sustained-domain profile. The interpretation developed below is deliberately organized at three evidential levels: (1) clinical observations directly documented in the present cases; (2) established physiological evidence from prior human and experimental work; and (3) a hypothesis-generating mechanistic proposal concerning the possible contribution of specific afferent populations. These levels should not be considered equivalent in evidential strength. The clinical heterogeneity of the four cases is intentional and should not be interpreted as evidence for a single underlying disorder or common pathological mechanism; rather, the temporal-domain framework is proposed as an interpretative model applicable across different vestibular phenotypes.

### 4.1. SVIN Is Not Redundant with vHIT

vHIT assesses the angular vestibulo-ocular reflex during brief high-acceleration head impulses. It provides a measure of canal-driven VOR gain and corrective saccades. SVIN, by contrast, is produced by bone-conducted skull vibration and reflects the effect of a high-frequency mechanical stimulus on vestibular end organs and afferent pathways. A normal or near-normal vHIT therefore does not exclude an abnormal SVIN: the two tests differ in stimulus type, temporal profile, receptor-afferent recruitment and output behavior. Importantly, relationships between SVIN and vHIT are not uniformly one-to-one in the literature; vestibular neuritis cohorts have demonstrated that vibration-induced responses can evolve differently from other vestibular measures during recovery [6,7].

### 4.2. SVIN Is Not Redundant with VEMPs

cVEMPs and oVEMPs assess specific otolithic-reflex pathways, mainly saccular-collic and utricular-ocular pathways, respectively [4]. SVIN, however, is not simply a nystagmographic equivalent of VEMPs. It is a directionally organized vestibulo-ocular response during skull vibration. Normal VEMPs do not exclude abnormal vibration-induced nystagmus because VEMPs and SVIN differ in output pathway, temporal dynamics and afferent weighting. Previous clinical work has already documented incomplete agreement between SVIN and otolithic-reflex asymmetry, supporting complementary rather than interchangeable interpretations of these measures [8,11].

### 4.3. SVIN and Velocity Storage

Classical velocity-storage-dependent responses are characterized by sustained vestibular dynamics, post-rotatory persistence, optokinetic after-nystagmus and prolonged temporal integration. SVIN has a different temporal signature: it is typically stimulus-locked, beginning rapidly with vibration onset and stopping rapidly at vibration offset [1,2]. The coexistence of abnormal velocity-storage-related tests and strong SVIN in some patients does not imply identity between these mechanisms. Instead, it emphasizes the need to distinguish fast vibration-sensitive asymmetry from sustained visuo-vestibular integration.

### 4.4. Temporal-Domain Dissociation

The classical clinical reading of vestibular tests often assumes an anatomical logic: vHIT for semicircular canals, cVEMP for saccular-inferior vestibular pathways, oVEMP for utricular-superior vestibular pathways and SVIN as a lateralizing sign of vestibular asymmetry. This remains useful, but it is incomplete. Earlier clinical work already emphasized the multifrequency nature of vestibular assessment and documented discordant vibration, caloric and head-shaking responses in partial vestibular lesions, particularly Ménière’s disease and vestibular neuritis [5,6,9]. The present temporal-domain interpretation therefore does not claim novelty for the existence of discordance itself. It adds a second, explicitly dynamic axis by considering each test as a probe of a different temporal regime: sustained, impulsive, vibratory, reflex, canal, otolithic or mixed canal-otolith.

**Figure 2:**
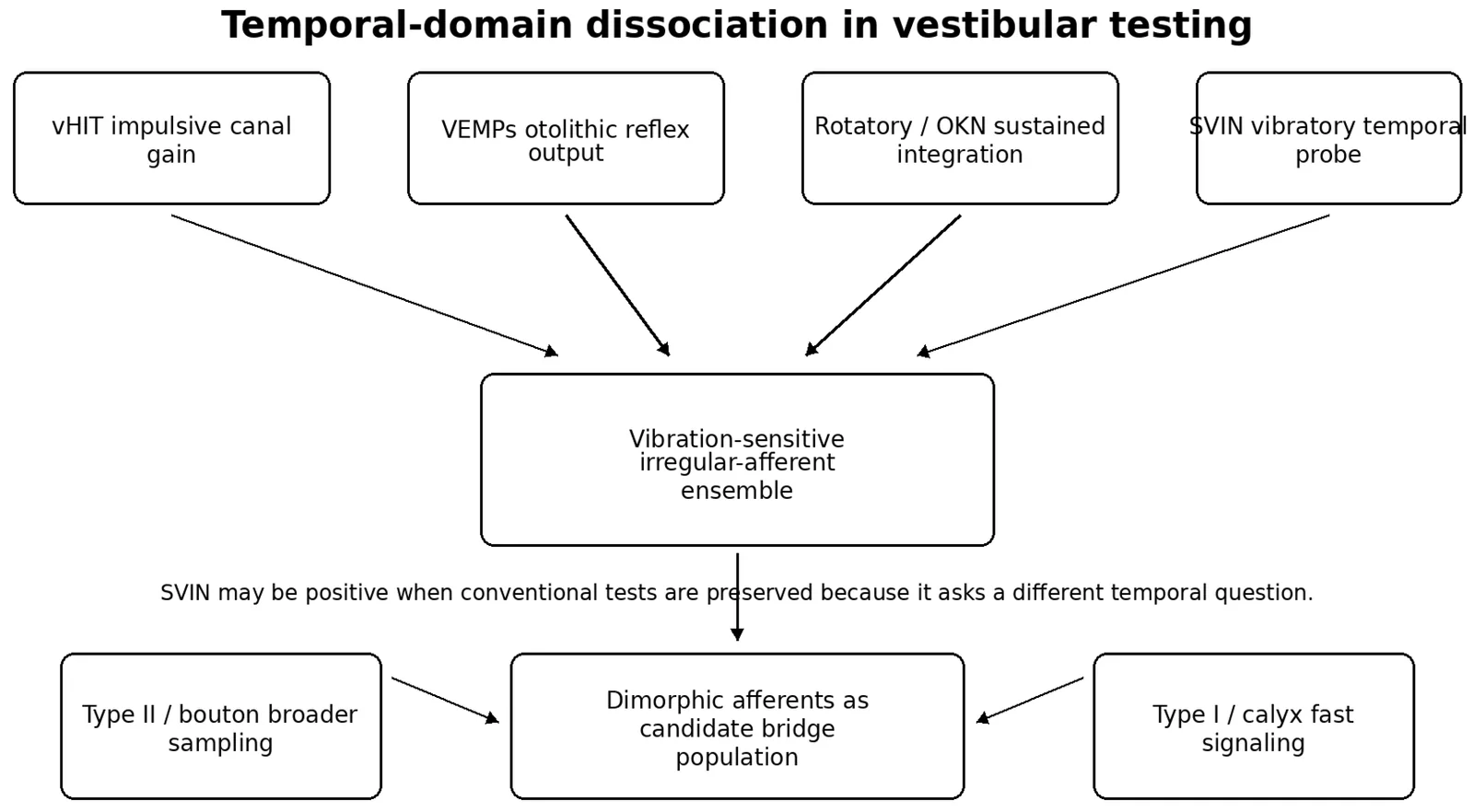
Conceptual model of temporal-domain dissociation in vestibular testing. The upper test-domain layer represents clinical observations and established test physiology: vHIT, VEMPs, rotatory/optokinetic paradigms and SVIN interrogate different dynamic modes of vestibular function. The middle irregular-afferent/vibration-sensitive layer represents established physiological evidence that 100-Hz bone-conducted vibration preferentially recruits irregular vestibular afferents and can generate phase-locked activity [11,13]. The lower dimorphic-afferent layer is explicitly hypothesis-generating: dimorphic afferents are proposed only as a candidate bridge population linking Type I/calyx high-bandwidth signaling with broader Type II/bouton receptor sampling, not as a proven or exclusive generator of SVIN. Arrows indicate the proposed direction of functional linkage between the test domains, the vibration-sensitive irregular-afferent ensemble, and the candidate dimorphic-afferent bridge population.

In this framework, discordance between tests may reveal not failure of logic, but separation between temporal channels and, in selected cases, abnormal central shaping of vibration-sensitive input. A patient may show normal vHIT and symmetric VEMPs yet a clear SVIN because high-acceleration canal gain and short-latency vestibulomyogenic outputs do not exhaust the vibration-sensitive vestibulo-ocular ensemble. Conversely, as illustrated by Case 4, a selective cVEMP abnormality may coexist with normal vHIT, symmetric oVEMPs and a clinically informative SVIN without the vibration-induced response simply duplicating the VEMP deficit. An atypical bilaterally evoked, multidimensional response whose horizontal/vertical weighting changes with the stimulated mastoid, as in Case 3, may further indicate central visuo-vestibular shaping of the final oculomotor output. This framework is summarized in Figure 2.

### 4.5. Physiological Foundation: Irregular Canal Afferents and Curthoys 1982

Curthoys recorded primary horizontal semicircular canal neurons in rats and guinea pigs during angular acceleration and divided them into regular and irregular categories according to the regularity of resting discharge [14]. This work sits within a broader physiological literature showing systematic diversity in resting discharge, sensitivity and dynamic behavior of first-order canal afferents across species [15,16,17,18]. Regular neurons tended to have higher average resting rates, lower sensitivity and longer time constants; irregular neurons tended to show higher sensitivity, shorter time constants and, in some cells, greater responsiveness to incremental than decremental accelerations of the same magnitude [14].

This is crucial for the present argument. Irregular canal afferents are not simply regular afferents with more gain. They display a distinct temporal phenotype: more phasic, more sensitive to acceleration dynamics and more capable of expressing rapid changes in rotational acceleration. This gives the canal system its own transient domain, parallel to the otolithic transient domain revealed by VEMPs and sound/vibration studies [3,4]. A broader multiscale interpretation of acceleration- and jerk-sensitive vestibular encoding has also been proposed [19].

### 4.6. Bone-Conducted Vibration, Irregular Afferents and Bypass of Velocity Storage

Experimental and clinical work has strengthened the view that bone-conducted vibration preferentially activates vestibular primary afferents with irregular resting discharge. Curthoys specifically summarized the neural basis of SVIN, including cycle-by-cycle phase locking of irregular canal afferents to 100-Hz vibration and the abrupt stimulus-locked onset and offset of the ocular response [11]. More recent work further supports the view that the neural response to vibration largely bypasses the indirect pathway and the velocity-storage integrator [2,13]. This framework explains the characteristic temporal profile of SVIN: abrupt onset at stimulus onset, maintenance during vibration, abrupt offset at stimulus termination and little or no after-nystagmus.

### 4.7. Type I-Calyx Synaptic Architecture and Ultrafast Signaling

Any hypothesis about a vibration-sensitive vestibular subchannel must explain how the periphery achieves high temporal precision. Simultaneous recordings from vestibular Type I hair cells and their calyceal afferents demonstrate multiple modes of transmission at these specialized endings, including conventional quantal transmission, slower potassium-mediated components and fast bidirectional electrical communication/resistive coupling at the narrow synaptic cleft [20,21]. This architecture provides a plausible biophysical basis for high-bandwidth vestibular signaling.

### 4.8. Why Dimorphic Afferents Matter

Dimorphic afferents are considered here as a hypothesis-generating anatomical candidate through which temporal-domain dissociation might become clinically visible. A dimorphic fiber can receive calyceal input from Type I hair cells and bouton input from Type II hair cells within the same afferent arbor. This dual receptor sampling makes dimorphic afferents biologically interesting as a potential interface between fast calyceal signaling and broader bouton-based receptor sampling [18,21,22]. It does not, however, establish a privileged or causal role in SVIN.

The present paper does not claim that SVIN is generated selectively by dimorphic afferents. The evidence does not support such an overstatement. Rather, the proposal is narrower: within the irregular, vibration-sensitive vestibular channel, dimorphic afferents may represent a plausible candidate bridge population capable of explaining persistent clinicophysiological dissociations. The final nystagmic phenotype, however, may also be shaped by central visuo-vestibular processing, particularly when the response is bilateral, contains a vertical component or is associated with marked oculomotor asymmetry. The temporal-domain roles of the principal vestibular tests are summarized in Table 2.

## 5. Proposed Hypothesis

Primary supported framework: bone-conducted vibration preferentially recruits irregular vestibular afferents and largely bypasses the indirect pathway and velocity storage. SVIN therefore acts mainly as a probe of a direct, irregular, vibration-sensitive vestibular channel rather than as a test of the velocity-storage system [2,13].

Second-level proposal: within this broader irregular channel, dimorphic afferents may represent a plausible bridge population capable of generating persistent clinicophysiological dissociations. This proposal rests on four considerations: (1) dimorphic afferents have dual receptor sampling by definition; (2) jerk-sensitive vestibular ensembles are not limited to calyx-only pathways but include calyx-rich and dimorphic irregular populations; (3) the Type I-calyx microcircuit possesses ultrafast signaling machinery; and (4) a dimorphic channel would be well suited to produce micro-asymmetries of recruitment rather than complete lesions.

Accordingly, persistent SVIN lateralization with otherwise preserved conventional vestibular metrics may reflect not a major canal loss or major otolith loss, but asymmetry within a mixed, jerk-sensitive, vibration-sensitive peripheral channel for which dimorphic afferents are a plausible candidate substrate.

## 6. Testable Predictions

First, there should be patients with reproducible SVIN lateralization across a spectrum of conventional findings, including normal or near-normal vHIT, symmetric VEMPs, or selectively dissociated cervical and ocular VEMP responses.

Second, such patients may show stability in SVIN asymmetry over time even when other vestibular metrics fluctuate little or normalize.

Third, refined physiological techniques may reveal abnormalities not as gross end-organ failure but as altered threshold, synchrony, phase-locking precision or transient recruitment within vibration-sensitive afferent populations.

Fourth, experimental models that distinguish calyx-only from dimorphic contributions to bone-conducted vibration-sensitive vestibular signaling may show that dimorphic populations contribute disproportionately to persistent asymmetric responses that outlast routine vestibular deficits.

## 7. Clinical Implications

The clinical implication is straightforward: a positive SVIN should not be dismissed as inconsistent or non-specific simply because vHIT is preserved, VEMPs are symmetric, or one otolithic-reflex output is selectively abnormal. In selected patients, SVIN may identify a hidden vestibular asymmetry expressed preferentially in the vibratory temporal domain. The morphology of the response must nevertheless be respected: a classic directionally stable horizontal response is not equivalent to an atypical bilateral response with a vertical component. The latter should prompt consideration of central or network-level visuo-vestibular modulation, as illustrated by the adolescent case.

SVIN should therefore be interpreted within a multidomain vestibular profile. Direction, stimulation-site dependence, horizontal, vertical and torsional components, quantitative slow-phase velocity, and coherence with oculomotor findings should all be considered. Its diagnostic value may lie precisely in revealing an asymmetric phase-locked signature that remains underrepresented by conventional canal, vestibulomyogenic, or sustained-domain measures.

## 8. Limitations

This study is a small retrospective, purposively selected, hypothesis-generating case series and was not designed to estimate prevalence, diagnostic sensitivity, or specificity. The patients were clinically heterogeneous, and not all paradigms or quantitative values were available in every case because testing followed clinical indication rather than a fixed research protocol. Measurements were therefore classified according to source status as contemporaneous, historical, or unavailable, and unavailable values were not inferred. Graph-derived eye-velocity estimates were not treated as definitive software measurements. The study cannot establish the cellular generator of SVIN; in particular, dimorphic afferents should be regarded as a candidate bridge population within the broader irregular, vibration-sensitive vestibular channel, not as an exclusive or proven substrate.

## 9. Conclusions

SVIN may disclose a “hidden” vestibular asymmetry or an atypical vibration-sensitive network response that is not adequately represented by the overall vHIT, SHIMP, VEMP, caloric, rotatory, optokinetic, or velocity-storage-related profiles. Here, “hidden” does not imply uniformly normal conventional testing. Across the four cases, the unifying feature was physiological non-congruence rather than uniform preservation of conventional tests.

The findings support SVIN as a distinct phase-locked clinical probe, preferentially weighted toward irregular-afferent signaling and requiring interpretation according to its morphology and multidomain context. Dimorphic afferents merit experimental investigation as a possible bridge between calyceal high-bandwidth signaling and broader bouton-based receptor sampling, but this proposal remains hypothesis-generating.

## Figures and Tables

**Table 1:** **Uniform cross-case clinical and physiological profile.** Data-source notation for Table 1: C = contemporaneous measurement; H = historical measurement; NA = measurement not available in the source clinical record. Historical data are retained for context but are not treated as evidence of preserved function at the contemporaneous examination.

Case	Clinical Phenotype	Bedside/HST	vHIT-SHIMP	VEMPs	Sustained/OKN Domain	SVIN Phenotype	Main Dissociation
1	Chronic instability and lateropulsion	No significant spontaneous NY; few HST beats; atypical positional downbeating/geotropic pattern	Horizontal HIMP/SHIMP substantially preserved; selective LARP reduction	H: cVEMP/oVEMP previously normal; no contemporaneous reassessment	Rotatory gain/time constants preserved; bilateral OKN reduction	Robust fixed horizontal response from both mastoids; mean SPV 4.9 degrees/s right and 3.5 degrees/s left stimulation; small vertical component	Disproportionate vibration-sensitive response relative to selective canal and OKN abnormalities
2	Persistent postural unsteadiness	No organized spontaneous NY comparable to SVIN; HST not available	C: normal six-canal vHIT; SHIMP NA	C: cVEMP and oVEMP bilateral and symmetric	Gain 0.27 at 0.22 Hz; OKN gain 0.03 bilaterally; post-rotatory constants 3.5/1.0 s	Exceptionally large fixed left-beating response from both mastoids; left stimulation adds upbeating component; no torsion	Intra-phase-locked dissociation: symmetric VEMPs vs. explosive SVIN; separate higher-order slow-domain abnormality
3	Adolescent recurrent vertigo with headache	No spontaneous/positional NY; HST negative	C: vHIT and SHIMP preserved and symmetric	C: bilateral; cVEMP AR 17%, oVEMP AR 25%	Rotatory gain 0.43; time constants 5.2/6.4 s; severe OKN asymmetry 0.35 leftward vs. 0.03 rightward	Site-dependent multidimensional SVIN: right mastoid mixed horizontal–vertical response (vertical peak slightly larger); left mastoid predominantly vertical with minimal horizontal activity; source-verified stimulation about 18–20 s	Atypical stimulation-site-dependent SVIN with preserved peripheral reflexes and central/network-level OKN abnormality
4	Persistent postural instability	C: no meaningful spontaneous NY; HST negative/low-velocity	C: normal six-canal vHIT; SHIMP NA	C: right cVEMP absent (AR 100%); oVEMPs bilateral and symmetric	C: reduced OKN; gain 0.18 leftward vs. 0.13 rightward at 40 degrees/s; rotatory/post-rotatory NA	C: site-dependent horizontal SVIN; left mastoid stronger (mean SPV −3.2/−2.7 degrees/s by eye) than right mastoid (−0.9/−0.8 degrees/s); stimulus markers about 10–11 s	Selective phase-locked dissociation: absent right cVEMP with symmetric oVEMPs and normal vHIT; quantitatively documented SVIN not reducible to a single conventional measure

**Table 2:** Vestibular tests as temporal-domain probes.

Test	Dominant Clinical Window	Temporal/Dynamic Question	Why Discordance May Occur
vHIT	Impulsive canal VOR gain	Can the canal-driven VOR generate appropriate eye velocity during brief high-acceleration head impulses?	Can be normal if gain pathways are preserved despite vibratory ensemble asymmetry.
cVEMP	Short-latency sacculo-collic pathway	Can a transient otolithic stimulus generate a cervical myogenic reflex?	Can be normal if the saccular reflex pathway is preserved despite canal-otolith vibratory asymmetry.
oVEMP	Short-latency utriculo-ocular pathway	Can a transient otolithic stimulus generate an ocular myogenic reflex?	Can be normal if utricular reflex output is symmetric while SVIN recruits mixed dynamic ensembles.
Rotatory/OKN/OKAN	Sustained visuo-vestibular integration	Can vestibular and optokinetic signals be temporally stored and integrated over seconds?	May be abnormal when slow central integration is impaired even if fast canal gain is preserved.
SVIN	Repetitive vibratory canal-otolith ensemble	Can the two labyrinths symmetrically process rapid repeated acceleration transients?	May be abnormal when dynamic high-bandwidth asymmetry is present despite normal vHIT and VEMPs.

## Data Availability

The anonymized clinical data underlying this article are available from the corresponding author upon reasonable request, subject to privacy and ethical restrictions.

## Supplementary Materials

The following supporting information can be downloaded at: https://www.mdpi.com/article/10.3390/audiolres16050123/s1, Supplementary Figure S1: Case 1—site-dependent horizontal SVIN; Supplementary Figure S2: Case 3—multidimensional, stimulation-site-dependent SVIN; Supplementary Figure S3: Case 4—site-dependent horizontal SVIN.

## Abbreviations

AR	asymmetry ratio
cVEMP	cervical vestibular-evoked myogenic potential
HIMP	head impulse paradigm
HST	head-shaking test
LARP	left anterior-right posterior canal plane
OKAN	optokinetic after-nystagmus
OKN	optokinetic nystagmus
oVEMP	ocular vestibular-evoked myogenic potential
PTA	pure-tone average
RALP	right anterior, left posterior
SHIMP	suppression head impulse paradigm
SPV	slow-phase velocity
SVIN	skull vibration-induced nystagmus
VEMP	vestibular-evoked myogenic potential
vHIT	video head impulse test
VNG	videonystagmography
VOR	vestibulo-ocular reflex
VORS	vestibulo-ocular reflex suppression
VVOR	visually enhanced vestibulo-ocular reflex
